# Whole Mind Body Psycho-Education Group– The Essence of MDT and Patients Participation Highlighting Holistic Psychiatry Learning Experiences and MDT Working to Achieve Better Outcomes for Patients, to Support with Patient's Recovery Journey

**DOI:** 10.1192/bjo.2026.11300

**Published:** 2026-06-30

**Authors:** Omer Malik, Sujata Sharma, Karin Dicander, Emma Hahn, Frederick Taylor

**Affiliations:** Cygnet Healthcare, London, United Kingdom

## Abstract

**Aims:**

Psychoeducation is a core component of recovery-focused inpatient mental health care, supporting service users to better understand their illness, treatment, and legal framework. The Whole Mind Group was developed to provide structured, accessible psychoeducation delivered collaboratively by a multidisciplinary team (MDT) within an inpatient setting.

The aim of the Whole Mind Group was to enhance patients’ understanding of their mental health, medication, legal status, and wellbeing through interactive, MDT-led psychoeducational sessions, and to evaluate participant satisfaction with the knowledge gained, its applicability, and group facilitation.

**Methods:**

The Whole Mind Group was delivered weekly on Mondays from 2:30 pm to 3:30 pm over a 16-week period, running from 12 May 2025 to August 2025, with two repeat cycles of six core topics: understanding medication, stress and trauma, affective disorders, psychosis, section and rights, and substance misuse. The group was open to patients acrossthree wards at Cygnet Churchill Hospital (two acute male wards and one male rehabilitation ward). Sessions were facilitated by the MDT, including consultant psychiatrist, occupational therapist, psychologist, mental health act lead, and assistant psychologists.Delivery was interactive, with emphasis on participation, shared learning, and maintaining a psychologically safe environment. Feedback was collected at the end of each session using a standardised questionnaire based on a 7-point Likert scale assessing satisfaction with practical knowledge received, ability to apply knowledge, and facilitation of the psychology group.

**Results:**

Participant feedback was analysed using a 7-point Likert scale (1–7) across three domains: knowledge, application of knowledge, and group facilitation. Mean scores were converted to percentages to support interpretation.

Overall scores were high across all domains. Knowledge and group facilitation both achieved a mean score of 6.04/7 (86.3%), while application of knowledge achieved a mean score of 5.46/7 (78%).

Across individual sessions, Understanding Affective Disorders received the highest overall score with a mean total of 19.33/21 (92%). The lowest overall score was recorded for Understanding Your Medication, with a mean total score of 15.18/21 (72.3%).

The overall mean total score across all sessions was 17.54/21, indicating consistently positive participant feedback regarding knowledge gain, applicability, and facilitation of the Whole Mind psychoeducation group.

**Conclusion:**

The Whole Mind Group demonstrates the value of structured, MDT-led psychoeducation within inpatient mental health services. The consistently positive feedback indicates that the group was well-received and supports its ongoing use as a collaborative, recovery-focused intervention promoting understanding, engagement, and shared learning among service users.

